# *Drosophila*-based screening of herbal compounds targeting FOXO signaling pathway in type 2 diabetes mellitus

**DOI:** 10.3389/fphar.2025.1621414

**Published:** 2025-07-31

**Authors:** Mukarram Mudjahid, Nadila Pratiwi Latada, Youdiil Ophinni, Firzan Nainu

**Affiliations:** ^1^Department of Pharmacy, Faculty of Pharmacy, Hasanuddin University, Makassar, Indonesia; ^2^Unhas Fly Research Group, Faculty of Pharmacy, Hasanuddin University, Makassar, Indonesia; ^3^The Hakubi Center for Advanced Research, Kyoto University, Kyoto, Japan; ^4^Center for Southeast Asian Studies (CSEAS), Kyoto University, Kyoto, Japan; ^5^Immunology Frontier Research Center (IFReC), Osaka University, Suita, Osaka, Japan; ^6^Center for Infectious Diseases (CID), Kobe University, Chuo-ku, Kobe, Japan

**Keywords:** *Drosophila*, Foxo, T2DM, herbal medicine, preclinical assay

## Introduction

Type 2 diabetes mellitus (T2DM) represents a multifactorial chronic metabolic disorder characterized by persistent hyperglycemia and associated with an escalating global prevalence (Ong et al., 2023). The pathophysiology of T2DM encompasses a network of molecular disturbances, including dysregulated glucose metabolism, heightened oxidative stress, and impaired insulin signaling (Galicia-Garcia et al., 2020; Mlynarska et al., 2025). These underlying mechanisms highlight the need for treatment strategies that target the root molecular pathways of this disease, beyond just glycemic control. The forkhead box O (FOXO) signaling pathway has become a crucial modulator of metabolic homeostasis in this regard. FOXO transcription factors regulate essential cellular processes such as glucose homeostasis, proliferation, apoptosis, and metabolic stress resistance (Wang et al., 2014; Teaney and Cyr, 2023). Dysregulation of this pathway has been linked to the progression and severity of diabetic complications.

Herbal medicine is increasingly recognized for its diverse bioactive compounds that simultaneously modulate multiple signaling pathways (Uti et al., 2025). However, a key challenge in the development of herbal drug candidates is the lack of detailed understanding of their specific molecular mechanisms of action, which frequently impedes their progress toward clinical application. Investigating these mechanisms typically necessitates the use of animal models, which allow for the complex interplay of signaling pathways to be observed in a physiological context. Nevertheless, conventional mammalian models are often constrained by high costs, long experimental timelines, and ethical concerns, limiting their practicality for large-scale mechanistic studies (Nainu et al., 2025). In this context, *Drosophila melanogaster* emerges as a valuable *in vivo* model system.

*Drosophila* possesses a highly conserved FOXO signaling pathway (FOXO-SP) that is orthologous to that of humans, making it a promising platform for translational studies on the effects of candidate drugs (Puig and Mattila, 2011). It supports precise genetic manipulation through its extensive mutant and transgenic resources, making it ideal for studying the interaction of herbal compounds with specific signaling pathways like FOXO (Lopez-Ortiz et al., 2023). Moreover, this model offers experimental efficiency for elucidating both the metabolic and molecular mechanisms of herbal compound activity (Lopez-Ortiz et al., 2023; Baenas and Wagner, 2019). In light of the considerable therapeutic potential, we propose a *Drosophila*–based high-throughput screening framework to evaluate herbal drug candidates, focusing on the FOXO-SP as a key regulatory node in T2DM. This approach may accelerate the discovery of active compounds and elucidation of their mechanisms, advancing evidence-based herbal therapeutics.

### FOXO-SP as a therapeutic target in the pathogenesis of T2DM

The FOXO-SP plays a crucial role in the regulation of cellular metabolism and stress responses, with significant implications in the development of T2DM (Wang et al., 2014). Among the FOXO family members in human, FOXO1 is particularly critical in the regulation of glucose and lipid metabolism (Teaney and Cyr, 2023). It has also been strongly implicated in the pathogenesis of T2DM (Teaney and Cyr, 2023; Marchelek-Mysliwiec et al., 2022). In T2DM, insulin resistance leads to reduced activation of the phosphoinositide 3-kinase/protein kinase B (PI3K/AKT) signaling. As a result, the diminished PI3K/AKT signaling fails to suppress FOXO1 activity, causing its accumulation in the nucleus and increasing its transcriptional activity (Batista et al., 2021). This activation of FOXO1 regulates various cellular responses across multiple tissues, including the liver, pancreas, skeletal muscle, and adipose tissue (Teaney and Cyr, 2023; Kousteni, 2012).

FOXO1 functions as a central transcriptional regulator of hepatic glucose metabolism. Under physiological conditions, insulin signaling via PI3K/AKT phosphorylates FOXO1, promoting its cytoplasmic retention and repressing gluconeogenic transcription in the liver (Teaney and Cyr, 2023). In T2DM, this regulation is impaired, thereby increases hepatic glucose production and contributes to chronic hyperglycemia (Zhang et al., 2006). Beyond glucose metabolism, FOXO1 also plays a pivotal role in modulating lipid metabolism and managing oxidative stress responses within hepatocytes. Its hyperactivation promotes the transcription of genes involved in lipolysis, such as adipose triglyceride lipase (ATGL) and hormone-sensitive lipase (HSL), leading to increased mobilization and systemic release of free fatty acids (Chakrabarti and Kandror, 2009). Elevated circulating free fatty acids further potentiate peripheral insulin resistance and contribute to lipotoxicity and β-cell dysfunction (da Silva Rosa et al., 2020).

FOXO1 also plays a complex role in skeletal muscle and adipose tissue. In skeletal muscle, FOXO1 influences both muscle mass and glucose metabolism. Elevated FOXO1 activity leads to muscle atrophy and a reduction in glucose uptake, especially under hyperglycemic conditions and when advanced glycation end-products (AGEs) accumulate (Teaney and Cyr, 2023; Giri et al., 2018). Furthermore, FOXO1 downregulates GLUT4 expression, worsening insulin resistance (Teaney and Cyr, 2023; Karnieli and Armoni, 2008). Studies on mutations in FOXO-SP have shown that inhibiting FOXO1 can enhance muscle mass (Vilchinskaya et al., 2022) and improve insulin sensitivity (Li et al., 2019). In adipose tissue, FOXO1 represses peroxisome proliferator–activated receptor gamma (PPARγ) transcription to inhibit adipogenesis. Excessive suppression of adipogenesis leads to ectopic fat accumulation in non-adipose tissues, such as the liver and muscles, contributing to lipotoxicity and insulin resistance (Peng et al., 2020).

### *Drosophila* as a model organism for screening herbal candidates against T2DM

*Drosophila melanogaster* has been extensively utilized as a model organism for investigating the molecular mechanisms underlying complex human diseases (Victor Atoki et al., 2025). It has significantly contributed to disease modeling in various contexts, such as neurodegenerative disorders (Nitta and Sugie, 2022), cancer (Bilder et al., 2021), infectious diseases (Harnish et al., 2021), and toxicological assessments (Leao et al., 2019). In recent years, the utility of *Drosophila* has extended to the study of metabolic disorders, where it offers unique advantages for dissecting the molecular underpinnings of metabolic dysfunction. Building on its established role in disease modeling, *Drosophila* has emerged as a powerful tool for investigating conserved pathways involved in metabolism.

Numerous studies have demonstrated conserved metabolic regulation between humans and *Drosophila*, particularly in insulin signaling, nutrient sensing, and energy homeostasis—core pathways in the pathophysiology of T2DM and obesity (Graham and Pick, 2017). Within this conserved signaling cascade, the FOXO-SP plays a pivotal role in metabolic regulation, and its impairment is closely linked to T2DM pathogenesis. Genetically, *D. melanogaster* contains a single FOXO gene, *dFOXO*, which serves as the exclusive evolutionary ortholog of the mammalian FOXO transcription factor family (Jünger et al., 2003). Compared to traditional vertebrate models, *Drosophila* offers advantages such as a rapid life cycle, cost-effectiveness, and advanced genetic tools for modeling disease (Victor Atoki et al., 2025). This is further supported by the extensive availability of mutant and transgenic strains, which significantly expands its applicability for *in vivo* functional genomics and targeted pathway analyses (Casas-Tinto, 2024).

These conserved features and experimental advantages have enabled the development of robust *Drosophila* models, which not only replicate T2DM-like phenotypes, but also facilitate the systematic investigation of underlying mechanisms and therapeutic interventions. For instance, silencing the expression of the PI3K catalytic subunit (Dp110) through RNA interference (RNAi) induces diabetes-like characteristics, such as hyperglycemia, reduced body size, and diminished glycogen stores (Mascolo et al., 2022). Mutations in the *InR* ^*E19*^ gene, an insulin receptor upstream of the FOXO pathway, also lead to disturbances in glucose and lipid metabolism that mimic diabetes (Murillo-M et al., 2011). The presence of signaling pathways that are homologous to those in mammals, combined with the availability of versatile genetic tools, makes *Drosophila* a powerful model system. This is relevant in the context of compound screening, especially for therapies targeting the FOXO-SP.

## Discussion

**FIGURE 1:**
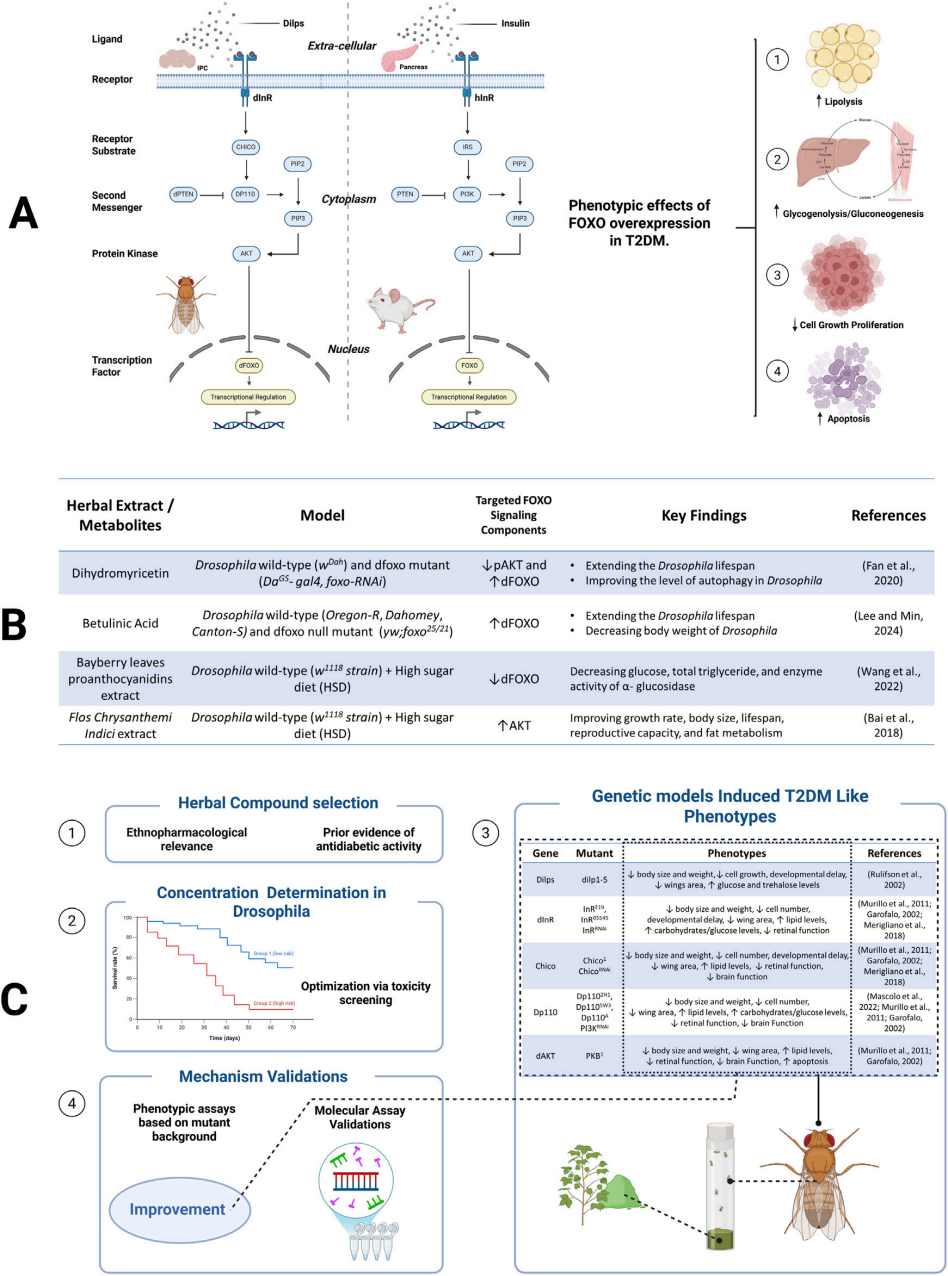
Overview of the FOXO-SP. **(A)** Conserved FOXO-SP in *Drosophila* and mammals, from ligand binding to FOXO regulation. **(B)** Herbal extract/metabolites modulate FOXO-SP. **(C)** A screening workflow to identify herbal compounds targeting the FOXO-SP.

Targeting the FOXO-SP in herbal compound screening for T2DM therapy offers several significant scientific advantages and practical applications. This contributes not only to poor glycemic control but also to the progression of diabetic complications such as nephropathy, neuropathy, and retinopathy (Mansour et al., 2023). Supporting this approach, previous studies have demonstrated that certain herbal compounds can modulate FOXO activity in Drosophila (see Figure 1B), indicating their potential to regulate FOXO-dependent signaling pathways (Wang et al., 2022, Bai et al., 2018, Rulifson et al., 2002, Garofalo, 2002, Merigliano et al., 2018). With FOXO gene orthologous to its human counterpart (see Figure 1A), *Drosophila* exhibits a rapid life cycle and a relatively small compound requirements for testing, making it a suitable model for high-throughput screening.

In this perspective, we emphasize the use of genetic *Drosophila* models that manipulate the components of FOXO-SP as representations of T2DM. Although non-genetic *Drosophila* models such as those induced by high-sugar or high-fat diets have also successfully recapitulated aspects of insulin resistance and metabolic dysfunction, genetic models offer significant advantages in phenotypic consistency, reproducibility, and the ability to directly investigate the molecular roles of specific pathways. This approach enables more detailed and efficient mechanistic studies, especially in screening drug candidates targeting specific components of the FOXO-SP. Moving forward, integrating diet-induced models could complement this perspective by enhancing clinical relevance in T2DM. To support the utility of these genetic models, the application of advanced molecular tools is essential for dissecting the FOXO-SP with high spatial and temporal resolution. The use of advanced genetic tools in *Drosophila*, such as the GAL4/Upstream Activating Sequence (GAL4/UAS system), RNAi, and reporter transgenics, enables precise manipulation of the *dFOXO* component pathways.

A systematic screening workflow was designed to implement this approach (see Figure 1C). Candidate compounds—including both purified phytochemicals and crude plant extracts—were selected based on their ethnopharmacological relevance and prior evidence of antidiabetic activity. Initial toxicity assays were recommended to identify sublethal and pharmacologically relevant dose ranges, thereby ensuring that observed phenotypic effects reflect bioactivity rather than toxicity. To mimic diabetic-like conditions, we can employ *Drosophila* mutants with impaired function in key components of the FOXO-SP. These mutants exhibit metabolic phenotypes analogous to T2DM, including reduced body size, impaired growth, elevated lipid and carbohydrate accumulation, and developmental delay (see Figure 1C). To evaluate therapeutic effects, herbal candidates could subsequently be administered via several routes (e.g., oral feeding or systemic injection). The phenotypic traits served as quantifiable readouts for assessing the metabolic effects of the candidate compounds. Interestingly, the same mutant models also facilitated mechanistic investigation by linking phenotypic improvements to modulation of the FOXO-SP. To validate this, RNA sequencing can be employed to see profile transcriptomic changes in response to treatment. This step enabling the identification of differentially expressed genes associated with the modulation of the FOXO-SP. By linking molecular insights to measurable phenotypes, this approach offers a methodologically robust and high-throughput platform that targets the FOXO-SP.

However, despite its prospects, several challenges remain in targeting the FOXO-SP and in utilizing *Drosophila* as a model organism. For example, FOXO activation has been associated with beneficial effects, such as lifespan extension and suppression of tumorigenesis in specific tissue contexts (Farhan et al., 2020; Fan et al., 2020; Lee and Min, 2024). These findings introduce complexity and raise concerns regarding the broad inhibition of FOXO, as therapeutic outcomes may vary depending on specific tissue and disease context. Therefore, understanding therapeutics goals and comorbid risks is essential when considering FOXO-targeted interventions. Moving forward, selective activation or inhibition of FOXO, when integrated with advanced drug delivery systems, may hold considerable opportunity in the optimization of T2DM therapy while minimizing off-target effects.

When employing *Drosophila* as a model, the evaluation of herbal compounds particularly in terms of solubility and pharmacokinetic profiling remains a challenge. The solubility of herbal compounds is a well-recognized consideration across all *in vivo* model organisms, as it directly impacts bioavailability, including in *Drosophila*. Notably, *Drosophila* requires only relatively low concentrations to elicit measurable biological responses due to its small body size and high sensitivity to compounds. This low-dose requirement can partially compensate for the limitations imposed by poor aqueous solubility. Furthermore, several organic solvents such as ethanol and DMSO have often been used in *Drosophila* at specific concentrations, making them suitable carriers for hydrophobic herbal constituents. However, preliminary studies are strongly recommended to optimize solvent selection and confirm non-toxicity before conducting experiments.

Regarding formulation and dosing for clinical translation, pharmacokinetic profiling evaluation in *Drosophila* is an emerging area. While the absence of a mammalian-like hepatic system limits direct comparison, *Drosophila* possesses mammalian-like xenobiotic detoxification systems, including cytochrome P450s, phase II enzymes, and transporters. These components support functional absorption, systemic distribution, and metabolism of small molecules. The feasibility of conducting pharmacokinetic and biodistribution analyses in *Drosophila* has been demonstrated in a recent study (Sadova et al., 2024), providing a foundation for its use in early-stage pharmacokinetic research. However, further characterization and the establishment of standardized dose-conversion metrics are needed to strengthen translational relevance. Nevertheless, *Drosophila* offers distinct advantages for early-phase drug discovery, including pathway-specific mechanistic elucidation, target identification, and detection of off-target effects within a whole-organism context. These features are particularly valuable for prioritizing bioactive compounds prior to validation in mammalian systems. To bridge the translational gap, a tiered experimental framework integrating *Drosophila* with complementary mammalian models and clinical data is warranted. Such an approach not only accelerates the identification of lead compounds but also reinforces the scientific rigor of preclinical development pipelines.
